# Effectiveness of matching human leukocyte antigens (HLA) in corneal transplantation: a systematic review protocol

**DOI:** 10.1186/s13643-021-01704-7

**Published:** 2021-05-20

**Authors:** Gagandeep Singh Sachdeva, Joshua Piollo Cabada, Syed Saad Karim, Dmitry Lakvin Kahandawa, Kevin Anil Thomas, Anusha Kumar, Robert J. Barry, Gibran Farook Butt

**Affiliations:** 1 Institute of Clinical Sciences, College of Medical and Dental Sciences, University of Birmingham, Edgbaston, Birmingham, B15 2TT UK; 2 Academic Unit of Ophthalmology, Institute of Inflammation and Ageing, University of Birmingham, Edgbaston, Birmingham, B15 2TT UK

**Keywords:** Systematic review, Meta-analysis, Corneal transplantation, Keratoplasty, Antigen matching, HLA matching, Rejection, Reaction, Failure, Survival

## Abstract

**Background:**

Corneal transplantation is the most frequently performed transplantation in the UK. Despite this, the therapeutic value of matching human leukocyte antigen (HLA) subtypes for transplanted corneas remains controversial. Ocular immune privilege was originally deemed to render matching unnecessary; however, more recently, matching has demonstrated improved outcomes including graft success, amongst others. This systematic review aims to evaluate the effectiveness of major and minor antigen matching on graft outcomes in corneal transplantation.

**Methods:**

Standard systematic review methodology will be used to identify, select and extract data from observational studies and clinical trials assessing the effects of HLA matching on corneal graft outcomes. Bibliographic databases (Cochrane Library, EMBASE, MEDLINE, Web of Science, Scopus), clinical trial registers, abstract and conference proceedings, in addition to dissertation, thesis and grey literature will be searched. Neither date of publication nor language will be restricted, and non-English articles will be translated where necessary. The primary outcome will be to assess corneal graft success for different degrees of HLA matching/mismatching. The precise end outcome measure varies amongst studies and includes graft rejection, immunoreaction, failure and survival. Therefore, data will be extracted across all relevant outcome parameters and grouped for subsequent statistical tests. Risk of bias assessment will be completed, appropriate to each study design. Study selection, data extraction and risk of bias assessment will be independently completed by two reviewers. Data will be tabulated, and a narrative synthesis presented. Meta-analysis will be performed where there is sufficient homogeneity between studies to warrant its effective completion. Subgroup and sensitivity analysis will be undertaken if appropriate.

**Discussion:**

Many studies have investigated the effectiveness of HLA matching for corneal transplantation. A systematic review is needed to collate and analyse this evidence. Findings of this systematic review may form the basis of evidence-based recommendations on pre-operative HLA typing and matching of corneal grafts for transplantation.

**Systematic review registration:**

PROSPERO reference CRD42020198882

**Supplementary Information:**

The online version contains supplementary material available at 10.1186/s13643-021-01704-7.

## Background

Amongst preventable and untreated causes, corneal blindness is regarded as a common cause of visual impairment worldwide [1, 2]. Corneal transplantation is the only effective sight-restoring intervention for corneal blindness. It is the most frequently performed transplantation in the UK with over 4000 procedures annually since 2016 [3, 4]. It is an intervention of unmet demand, with disproportionately low supplies of donor corneas available in countries with the highest rates of corneal transplantation [5]. This disparity has detrimental consequences for a treatable population of patients with corneal blindness.

The most common indications for corneal transplantation in the UK remain keratoconus, pseudophakic bullous keratopathy, Fuchs’ endothelial dystrophy, infection, and graft failure [4]. There are various techniques for corneal transplantation, and the field has observed significant advances over recent decades. While the indication determines the precise type of transplantation procedure, full thickness and anterior lamellar grafts constitute over a third of the corneal transplants performed in the UK [4].

Corneal transplantation is regarded as one of the most successful transplantation procedures attributed principally to the cornea being an immunologically privileged site [6]. Despite the relatively lower immunogenicity of the cornea and the use of postoperative prophylactic treatment, 20 to 30% of corneal transplant patients still experience at least one episode of acute rejection within the first 5 years [7, 8]. In cases where acute rejection is irreversible, graft failure may ensue.

Rejection and graft failure are more commonly observed amongst high-risk corneal transplants. A number of high-risk factors are reported in the literature with the most well-recognised being underlying ocular surface inflammatory conditions, re-transplantation, corneal neovascularization and neolymphangiogenesis, glaucoma, previous ocular surgery and male to female transplantation [9]. In these high-risk cases, rejection can occur in 30 to 60% of grafts, with up to a 70% graft failure rate within 10 years—despite local or systemic immunosuppressive therapy [10–12].

Human leukocyte antigen (HLA) matching is recommended for other organ transplantations to offer the best opportunity for graft success [13–15]. However, the evidence supporting HLA typing for corneal transplantation remains less clear, with no international consensus.

Scoping of the literature revealed a summary document for a 1995 systematic review, which pooled results from 8 studies from 1966 to 1995, concluding that there was a non-significant effect of HLA-DR mismatching on first graft rejection: RR −0.13 (95% CI −0.35, 0.09) [16]. However, specific questions about the methodology, including study selection and analysis, were indeterminable from this report.

More recently, a 2015 narrative review discussed the value of major and minor HLA matching on corneal transplantation outcomes. They included thirty studies from 1974 to 2006 and concluded that despite controversial results being presented in older studies, recent evidence suggests HLA matching is beneficial for corneal allograft survival in general and even more significantly in high-risk allografts [17]. However, this conclusion was based on common themes amongst the outcome data, and standard systematic review methodology was not used.

HLA matching does not form part of the current corneal allocation policy in the UK [3]. Considering recent work within this active field, the proposed systematic review aims to evaluate the existing literature to determine the effect of HLA matching on corneal transplantation success. This may form the basis of evidence-based recommendations for future clinical practice.

The objective of this study will be to assess the effectiveness of HLA matching for corneal transplantation through the completion of a systematic review of the studies:

- Assessing the effect of matching major HLA antigens on graft outcomes
- Assessing the effect of matching minor HLA antigens on graft outcomes

## Methods

### PROSPERO registration

The protocol for this systematic review was registered with the PROSPERO database (reference CRD42020198882) [18]. The review and its findings are reported in accordance with the PRISMA guidelines [19]. A PRISMA-P checklist for this protocol is shown in Additional file 1.

### Information sources and search strategy

The following electronic databases will be searched from their inception onwards:

- The Cochrane Library (CENTRAL Register of Controlled Trials)
- EMBASE
- MEDLINE, MEDLINE in process (Ovid)
- Web of Science
- Scopus

Registers of clinical trials

- WHO International Clinical Trials Registry Platform (ICTRP) (https://www.who.int/ictrp/)
- European Clinical Trials Database (EudraCT)
- Clinicaltrials.gov (www.clinicaltrials.gov)
- International Standard Randomised Controlled Trials Number Database (ISRCTN)
- UK Clinical Research Network (www.ukcrn.org.uk)

Abstract and conference proceedings:

- Conference Proceedings Citation Index (Web of Science)
- British library ZETOC

Dissertations, theses and grey literature:

- ProQuest (www.proquest.com)
- OpenGrey (www.opengrey.eu)
- British Library Ethos

For bibliographic databases, the search strategy will combine index and free terms for the surgical procedure and distinctive lamellar techniques.

A sample strategy from MEDLINE has been formulated to collate all relevant evidence, and this has been included as Additional file 2. For each of the databases above, the search strategy may be adapted as deemed appropriate. An iterative manner will be applied to complete the search from these sources. The bibliographic references of the 1995 systematic review and any appropriate evidence reviews will be hand searched to ensure that no relevant primary study has been missed. Furthermore, a clinical expert will be contacted to ensure no similar systematic reviews are currently ongoing. To collate a comprehensive range of evidence, no restrictions will be placed by either publication date or language. RefWorks and Rayyan will be used to manage the search results. This will also enable exclusion of any duplicate entries, study details, and references. Grey literature will also be searched alongside electronic databases to reduce the risk of publication bias being introduced into the systematic review.

#### Selection criteria

The following criteria will be utilised to select studies for this review.

- PICO framework (Table 1)
- Study design
  - RCTs, non-RCT trial-based studies and cohort studies.

**Table 1:** PICO framework used to generate this review

Population	Patients (humans) undergoing corneal transplantation
Intervention	Donor-recipient HLA matching
Comparator	Patients receiving unmatched/selectively matched/randomly allocated donor corneas
Outcome	Occurrence of rejection and failure

### Participants

- Patients of any age, gender or ethnicity undergoing any form of corneal transplant. No restriction on date of transplantation will be applied.

- Intervention and comparator
  - Comparing the use of major antigen matching to antigen mismatching.
  - Comparing the use of minor antigen matching to antigen mismatching.
- Outcomes
  - Primary outcome
    - Corneal graft prognosis in the postoperative period: number of graft rejections, immunoreactions, failure and/or survival

With the exception of limbal, endothelial and tectonic transplants, all type of corneal transplant will be analysed.

Major antigen studies will be defined as those that discuss the effect of MHC class I (HLA-A, HLA-B and HLA-C), class II (HLA-DP, HLA-DQ and HLA-DR) and/or class III genes [20]. As there is an abundance of minor antigen sub-types [21], any studies including histocompatibility complexes not concerning the aforementioned antigens will be considered as minor.

All types of corneal transplant will be eligible for inclusion regardless of the underlying disease it was used to treat. However, it is important to note that grafts for keratoconus and other non-inflammatory conditions are likely to have better outcomes, compared to outcomes in patients with inflammatory diseases and re-grafts. The analysis of the studies may therefore be grouped based on the underlying disease, should the studies permit such stratification. Studies that include both the assessment of major and minor antigen matching will also be included in this review.

### Selection process

Selection of studies will be in two stages:

1. Abstracts and titles of each study will be screened to exclude unnecessary data.
2. Potentially relevant studies will have their full texts extracted and assessed against the selection criteria.

The appropriateness of articles will be assessed independently by two reviewers (JPC and SSK). A third reviewer (GB) will resolve any conflicts of opinion between each assessment. This process will be outlined through a PRISMA flow diagram. Exclusion of studies will be recorded and discussed in this review, and any non-English language studies will be translated to allow for a fuller inclusion of relevant studies.

### Data extraction

Relevant data from the suitable studies will be extracted independently by two individual authors. Any differences in opinion will be settled by discussion between both authors. If insufficient, this will be followed by a referral to a third author to resolve the matter at hand. A standardised data collection form in Microsoft Excel will be created and used by the authors to summarise the extracted data. The study authors and publishing bodies may be contacted via email if any relevant information is missing from the reviewed studies, with three attempts made to retrieve the missing information with each 2 weeks apart.

For each study, the following information, but not limited to, will be extracted:

- Study characteristics
  - Authors, publication year, title and journal
  - Study design
  - Setting/location
  - Sample size
  - Length of follow-up and variability in postoperative treatment
  - Analysis
- Participant characteristics
  - Patient selection and recruitment criteria
  - Demographic data number, age, gender, socioeconomic status and ethnicity
  - Past ocular history
- Intervention and comparator
  - Donor-recipient HLA matching
  - Comparator: patients receiving unmatched/randomly allocated donor corneas
  - Any differences in underlying care between the treatment groups
- Outcomes and findings
  - Number of graft rejections/reactions/failures at pre-defined follow-up intervals
  - Graft survival times (time to rejection and time to failure)
  - Adverse events, if adequately reported in the studies: including side effects and complications of surgery or pre/postoperative medications, infections, dry eye or procedural risks (including immunosuppression pre/postoperative where appropriate)
  - Precision and statistical test results for each outcome
  - Completeness of follow-up for each outcome

### Quality assessment

The quality of all the included studies will be assessed independently by two reviewers. Any disagreements will be resolved by discussion between the two individuals. If necessary, a third reviewer will act as an impartial mediator. RCTs will be assessed using the Cochrane Handbook Risk of Bias tool (RoB 2) [22]. This will also be used to assess non-randomised trials; hence, it is understood that the criteria present in this tool for allocation concealment and randomisation will not be relevant. Any prospective controlled observational studies will be assessed using the guidelines present in Chapter 13 of the Cochrane Handbook [22]. The RoB 2 tool may also be used as a minimum assessment—again without all criteria of the tool being relevant for this type of study. In prospective observational studies, the most relevant information to evaluate is the group selection criteria, differences in patient characteristics, losses to follow-up, biases and other confounders. The assessment of the biases present in each of the included studies will be collated in a findings table. In particular, the authors acknowledge that there may be variation in the definition of the study endpoints across the literature, and this will, therefore, be interrogated as a source of bias.

### Data synthesis

Included studies will be grouped based on the type of corneal transplant used (intervention) and the outcome parameters measured. It may be necessary to further stratify the studies based on the underlying disease state. The data will be tabulated, and a narrative synthesis of the relevant evidence compiled for each outcome of relevance to the review. This will aid with the summary of findings from each study and help to identify patterns in the data. Bias within studies will be quantitatively assessed and tabulated using risk of bias tools.

If feasible and appropriate, outcome data will be used to perform random effects meta-analyses because heterogeneity is expected a priori. The random effects model assumes the study level effect estimates follow a normal distribution, considering both within-study and between-study variation [23].

Data pooling will be carried out for the purpose of generating a Forest Plot, which will serve as a visual representation of the pooled effect of the said data. However, data from studies with variable study designs will not be pooled together.

Where heterogeneity is significant in studies, subgroup analysis may be conducted in order to investigate the source of heterogeneity, if the completeness of a study’s data collection and reporting allows for this. Of note, the population studied includes patients (humans) undergoing corneal transplantation, both paediatric and adult patients. Subgroup analysis of these two cohorts may be performed, should this be deemed appropriate.

Given the high variability in follow-up periods across the studies to be analysed, time-specific data from the postoperative period might have to be grouped where appropriate. Alternatively, outcome data may be grouped across all postoperative periods if better suited.

### Reporting

The results of this systematic review and associated meta-analysis will be reported using guidance laid out by the PRISMA 2020 statement [19]. This will be done with the intention of ensuring the reporting of results is both complete and transparent, under a well-recognised checklist.

The robustness of the review methodology will be discussed. An examination of both the internal and external validity of the results will also be conducted, for a complete picture of the integrity of the evidence base on which this review will be founded.

Following this, the implications of this review for future research, practice, health guideline revision and implementation will be considered.

## Discussion

Immunological compatibility, as with other tissue grafts, may have a beneficial effect for improving corneal graft prognosis following transplantation. However, the current lack of consensus between existing evidence has led to the absence of HLA matching for corneal grafts. This review will comprehensively and systematically extract published evidence from a multitude of sources. This protocol will be the first of its kind to be published, and the first to be registered prospectively.

Any amendments made to this protocol when conducting the review will be outlined in PROSPERO and reported in the final manuscript, as appropriate.

We do not anticipate any operational issues to arise when performing the study. Practically, we are aware that many single-centre studies have investigated the role of HLA matching on corneal graft outcomes, with their own definitions of the graft outcome. Accounting for this, we anticipate the following:

- A large amount of numerical data will be extracted which may prove time intensive to collate formally to yield overall statistics.
- Given heterogenous definitions of graft outcomes between studies, criteria for study inclusion in sub-group analysis/overall pooling of studies may have to be defined further.

The aim of this systematic review will be to reach an overall verdict regarding the effects of antigen matching of corneal grafts on survival, ultimately to aid with the improvement of patient outcomes.

However, potential limitations both at the study level and review level are anticipated, which may affect the overall merit of any conclusions reached. At a study level, preliminary screening highlighted that many studies investigating the role of HLA matching for corneal grafts are dated, and therefore, their study designs and clinical methodology will have to be evaluated against the standards expected in current practice. The reliability of different methods of HLA typing will also have to be considered. At a review level, as discussed within the practical issues, study heterogeneity in definitions of graft outcomes may affect the quality of pooled data.

## Supplementary Information

**Additional file 1.** PRISMA-P 2015 Checklist

**Additional file 2.** Ovid Embase sample search strategy

## Data Availability

Not applicable.

## Abbreviations

HLA: Human leukocyte antigen
MHC: Major histocompatibility complex
PICO: Problem/Patient/Population, Intervention/Indicator, Comparison, Outcome
EMBASE: Excerpta Medica Database
CENTRAL: Cochrane Central Register of Controlled Trials
MEDLINE: Medical Literature analysis and Retrieval System Online
PRISMA: Preferred Reporting Items for Systematic Reviews and Meta-analyses
RCT: Randomized controlled trial
