# Supplementary figures and images for: Effectiveness of matching human leukocyte antigens (HLA) in corneal transplantation: a systematic review protocol
Source: Syst Rev. 2021 May 20;10:150. doi: 10.1186/s13643-021-01704-7 (PMC8136040; doi:10.1186/s13643-021-01704-7)

**
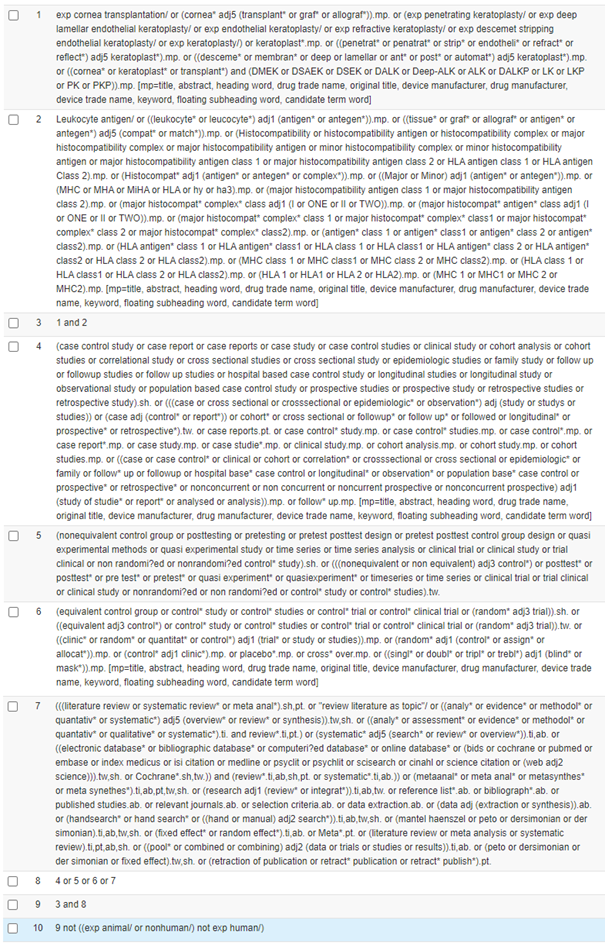
Ovid Embase sample search strategy**

Supplement: Supplementary file 2 — Additional file 2. Ovid Embase sample search strategy [file 13643_2021_1704_MOESM2_ESM.docx]
